# Limited Impact of Human Cytomegalovirus Infection in African Infants on Vaccine-Specific Responses Following Diphtheria-Tetanus-Pertussis and Measles Vaccination

**DOI:** 10.3389/fimmu.2020.01083

**Published:** 2020-06-05

**Authors:** Momodou Cox, Jane U. Adetifa, Fatou Noho-Konteh, Jainaba Njie-Jobe, Lady C. Sanyang, Abdoulie Drammeh, Magdalena Plebanski, Hilton C. Whittle, Sarah L. Rowland-Jones, Iain Robertson, Katie L. Flanagan

**Affiliations:** ^1^Infant Immunology Group, Vaccines and Immunity Theme, MRC Unit, Fajara, Gambia; ^2^School of Health & Biomedical Science, RMIT University, Melbourne, VIC, Australia; ^3^Department of Immunology and Pathology, Monash University, Melbourne, VIC, Australia; ^4^Department of Clinical Research, London School of Hygiene and Tropical Medicine, London, United Kingdom; ^5^Nuffield Department of Medicine, University of Oxford, Oxford, United Kingdom; ^6^School of Medicine and School of Health Sciences, University of Tasmania, Launceston, TAS, Australia

**Keywords:** human cytomegalovirus, human infants, measles vaccination, diphtheria-tetanus-pertussis (DTwP) vaccination, vaccine responses, antibodies, cellular immunity

## Abstract

Human cytomegalovirus (HCMV) infection has a profound effect on the human immune system, causing massive clonal expansion of CD8, and to a lesser extend CD4 T cells. The few human trials that have explored the effect of HCMV infection on responses to vaccination are conflicting, with some studies suggesting no effect whilst others suggest decreased or increased immune responses. Recent studies indicate substantial differences in overall immune system reactivity to vaccines based on age and sex, particularly cellular immunity. 225 nine-month old Gambian infants were immunized with diphtheria-tetanus-whole cell pertussis and/or measles vaccines. HCMV infection status was determined by the presence of CMV DNA by PCR of urine samples prior to vaccination. The effect of HCMV infection on either protective antibody immunity or vaccine-specific and overall cellular immune responses 4 weeks post-vaccination was determined, further stratified by sex. Tetanus toxoid-specific antibody responses were significantly lower in HCMV+ infants compared to their HCMV- counterparts, while pertussis, diphtheria and measles antibody responses were generally comparable between the groups. Responses to general T cell stimulation with anti-CD3/anti-CD28 as well as antigen-specific cytokine responses to purified protein derivative (PPD) were broadly suppressed in infants infected with HCMV but, perhaps surprisingly, there was only a minimal impact on antigen-specific cellular responses to vaccine antigens. There was evidence for subtle sex differences in the effects of HCMV infection, in keeping with the emerging evidence suggesting sex differences in homeostatic immunity and in responses to vaccines. This study reassuringly suggests that the high rates of HCMV infection in low income settings have little clinically significant impact on antibody and cellular responses to early life vaccines, while confirming the importance of sex stratification in such studies.

## Introduction

Human cytomegalovirus (HCMV) is a highly prevalent herpes virus (human herpes virus 5) that leads to lifelong chronic latent infection in humans once it is acquired. Latent infection is generally asymptomatic although symptomatic reactivation can occur, particularly in the immunocompromised or pregnant host (1, 2). The overall global prevalence is estimated at 83%, approaching 100% in developing countries (3) but closer to 66% in Europe (4). It tends to be acquired in adult life in developed countries while the epidemiology is quite different in resource poor settings where the majority of children will be infected before their first birthday (5).

HCMV infection is known to cause a massive clonal expansion of the CD8+ T cell population in adults (6) and children (5), and a lesser but still considerable expansion of CD4+ T cells in both adults and children (7) leading to inversion of the normal CD4:CD8 ratio to <1. The expanded T cells are typically terminally differentiated, as indicated by their lack of expression of CD27 and CD28 and positive expression of CD57 (5, 8). The combination of chronic HCMV infection, late-differentiated T cells and inverted CD4:CD8 T cell ratio and are all components of an “immune risk profile” (IRP) that has been associated with immunosenescence, cognitive decline, frailty and early death in the elderly (9–13). However, not all studies support these associations, for example HCMV infection was not associated with frailty in the largest longitudinal study conducted in the elderly to date (Newcastle 85+ Study) (14) and the IRP is not consistent across all populations studied and varies according to biological sex (15). Systemic inflammation, characterized by high serum levels of C reactive protein (CRP), tumor necrosis factor alpha (TNF-α) and interleukin 6 (IL-6), is also part of the IRP. It has been proposed that HCMV infection drives the inflammation associated with aging, so called “inflammaging” (16), but other studies refute such an association (17).

There is some weak evidence that HCMV infection may impair antibody responses to influenza vaccination in the elderly (18), but the studies are inconsistent and inconclusive and this question is yet to be resolved (19–21). A study of 263 18–52 year olds found no effect of HCMV infection on responses to a H1N1 pandemic influenza vaccine (22), while another found significantly lower responses in the HCMV+ as compared to the HCMV- cohort (18). Influenza vaccinated HCMV seropositive individuals >65 years of age had impaired *in vitro* inducible granzyme B release to influenza virus, but there was no apparent effect on antibody responses to standard titer or high titer influenza vaccination according to HCMV status (23). A systems biology analysis of younger (20–30 years) and older (60–>89 years) individuals found enhanced antibody responses following influenza vaccination in the HCMV+ healthy young adults compared to the HCMV- younger individuals (24). By contrast, the older adults had poorer responses to vaccination regardless of HCMV status. In the same study, murine CMV infected young mice were better protected against influenza challenge than their uninfected counterparts. Taken together this would suggest that HCMV enhances immunity in young healthy individuals.

A Gambian study showed that HCMV+ infants had similar antibody responses to measles, tetanus and *Haemophilus influenzae* b (Hib) vaccination at 13 and 18 months of age as compared to their HCMV- counterparts; and while they had lower CD4+ IFN-γ responses to measles 1 week after vaccination, there was no effect on measles-specific immunity 4 months post-vaccination (8). The HCMV+ infants did however have greater proliferation to polyclonal T cell stimulation with staphylococcal enterotoxin B, supporting enhanced cellular immunity in the HCMV infected children (8). Another study in 178 Gambian infants found that HCMV infection had no effect on antibody responses 2 months after measles and meningococcus A and C vaccination at 9 months of age (25). In the same study, EBV infection was associated with lower responses to all three vaccine antigens, and HCMV co-infection negated this negative effect on measles but not meningococcal antibody responses. This suggests a beneficial immunological effect of HCMV infection on T cell-dependent but not the T cell-independent vaccines (25). Similarly, a more recent longitudinal study of healthy children at 2, 5 and 10 years of age showed that EBV but not HCMV infection was associated with an accelerated decline in rubella and measles-specific IgG levels (26).

The effect of high rates of HCMV infection in infants in resource poor settings undergoing routine vaccination is therefore still not clear. Herein, we analyzed the effect of HCMV infection on antibody and cellular responses to diphtheria, tetanus-whole cell pertussis (DTP) vaccine and measles vaccine (MV) in a prospective study of 302 Gambian infants. Due to the well-described sex differences in immunity (27, 28) and vaccine responses (29) and the large cohort size and well-defined nature of the participants in this study, we were further able for the first time to analyse the effect of the infant's biological sex on the above parameters, with the aim of shedding light into potentially contradictory previous results.

## Materials and Methods

### Study Cohort and Vaccines Given

This is a sub-study nested within a randomized trial investigating the immunological effects of vaccination with diphtheria-tetanus-whole cell pertussis (DTP) vaccine, measles vaccine (MV) or both vaccines given together to 9 month old infant Gambians, the primary findings of which have been published previously (30, 31). We investigated the effect of HCMV infection on responses to the two vaccines. The main study recruited 302 four-month old infants presenting for routine vaccination at Sukuta Health Center, a peri-urban area 20 km from the coast of The Gambia. Eligibility criteria required that the child was healthy, had no history of chronic illness, no fever (<37.5°C), normal weight-for-age, and up to date with all recommended vaccines according to the national Expanded Program on Immunization (EPI) schedule. HIV status was not determined but the adult prevalence at the time of the study was low, estimated at between 1 and 4%, and would therefore be extremely low in these infants (5). Infants were randomized into one of three vaccine groups which determined the vaccines to be given at 9 months of age: Group 1 received a single standard intramuscular (i.m.) MV (Edmonston Zagreb strain, Serum Institute of India Ltd., Pune, India); group 2 received MV and i.m. DTP in two different sites (Serum Institute of India Ltd.); and group 3 received DTP alone (Table 1). Males and females were randomized separately. Groups 2 and 3 had their third dose of DTP withheld at 4 months to be given at 9 months, whereas group 1 received it as normal. All three groups received oral polio vaccine (OPV) and *Haemophilus influenza b* vaccine (Hib) at 4 months of age as recommended by the EPI. A 4.5 mL sample of venous blood was collected into a heparinised tube (7.5 units heparin/mL) at 9 months immediately prior to vaccination and 4 weeks later (median 28 days, interquartile range 28–29 days). Of the 302 infants recruited at 4 months, 286 received the study vaccine intervention at 9 months of age and 254 returned for follow-up at 10 months of age. Of these, 225 who had HCMV results and vaccine antibody and/or cytokine data are included in this sub-study (Table 1).

Table 1Study design including vaccines given from birth and infant numbers by HCMV status and sex.

**Table:** 

**Age**	**MV group**			**MV+DTP group**			**DTP group**		
Birth	BCG/OPV/HBV			BCG/OPV/HBV			BCG/OPV/HBV		
1m	OPV			OPV			OPV		
2m	DTP1/Hib/OPV/HBV			DTP1/Hib/OPV/HBV			DTP1/Hib/OPV/HBV		
3m	DTP2/Hib/OPV			DTP2/Hib/OPV			DTP2/Hib/OPV		
4m	**DTP3**/Hib/OPV/HBV			Hib/OPV/HBV			Hib/OPV/HBV

**Table:** 

9m	Blood sample, Urine for HCMV **MV** (*n* = 82)			Blood sample, Urine for HCMV **MV+DTP3** (*n* = 90)			Blood sample, Urine for HCMV **DTP3** (*n* = 53)		
		**HCMV–**	**HCMV+**		**HCMV–**	**HCMV+**		**HCMV–**	**HCMV+**
	**M**	22 (47.8%)	24 (52.2%)	**M**	12 (26.7%)	33 (73.3%)	**M**	7 (26.9%)	19 (73.1%)
	**F**	11 (30.6%)	25 (69.4%)	**F**	13 (28.9%)	32 (71.1%)	**F**	7 (25.9%)	20 (74.1%)
	**All**	33 (40.2%)	49 (59.8%)	**All**	25 (27.8%)	65 (72.2%)	**All**	14 (26.4%)	39 (73.6%)
10m	Blood sample, Urine for HCMV			Blood sample, Urine for HCMV			Blood sample, Urine for HCMV		
		**HCMV–**	**HCMV+**		**HCMV–**	**HCMV+**		**HCMV–**	**HCMV+**
	**M**	8 (17.4%)	38 (82.6%)	**M**	3 (6.7%)	42 (93.3%)	**M**	4 (15.4%)	22 (84.6%)
	**F**	4 (12.5%)	32 (87.5%)	**F**	6 (13.3%)	39 (86.7%)	**F**	1 (3.9%)	26 (96.1%)
	**All**	12 (14.6%)	70 (85.4%)	**All**	9 (10%)	81 (90%)	**All**	5 (9.4%)	48 (90.6%)

*Infants were recruited at 4 months of age and randomized to one of the 3 vaccine groups: The MV group received their normal 4 month vaccines and MV alone at 9 months; the other 2 groups had DTP3 delayed and either given with MV at 9 months (MV+DTP group) or given alone (DTP group). All infants received their recommended vaccines from birth. At 9 and 10 months of age blood samples were taken for antibody and cellular assays and urine tested for HCMV by PCR. The number of HCMV+ and HCMV- infants at 9 and 10 months are shown by sex and the percentage values indicate the percent HCMV- or HCMV+ in that vaccine group for males (M), females (F) or all infants combined (All). BCG, bacille Calmette-Guérin vaccine; OPV, oral polio vaccine; HBV, hepatitis B vaccine; DTP, diphtheria-tetanus-whole cell pertussis vaccine; Hib, Haemophilus influenzae type b vaccine. The bold letters indicate the vaccine interventions in the study protocol*.

### Ethics Statement

The study protocol was approved by the Joint Gambia Government/MRC Ethics Committee (project number SCC1085) and the London School of Hygiene and Tropical Medicine Ethics Committee. Written/thumb-printed informed consent was provided by a parent/guardian of all participating infants in accordance with the Declaration of Helsinki.

### Diagnosis of HCMV Infection

The infants were too young for serum HCMV IgG testing due to likely persistence of maternally-derived antibody. Therefore, urine samples were collected at 9 and 10 months of age and tested for the presence of HCMV DNA by a nested polymerase chain reaction (PCR) with a cut off of approximately 25 DNA copies/mL, as previously described (32, 33). Appropriate positive and negative controls were employed to reduce the likelihood of false positive diagnoses. Furthermore, infants that tested positive at 9 months were all confirmed to be positive at 10 months and are thus likely true positives since urinary secretion usually persists for at least 6 months post-infection. Urine was not available for all donors and only those with a result at 9 months of age are included in this study.

### Vaccine Antibody Assays

Heparinised whole blood was spun at 2,000 rpm for 5 min and plasma collected and stored at −70°C. The measles IgG haemagglutination inhibition assay (HAI) was performed using monkey red blood cells as previously described (34). Results are expressed as log_2_ units, the minimum detection level being 31.2mIU, and a protective level defined as ≥125mIU (log_2_ titer ≥3). A multiplex microsphere based fluorescent immunoassay for IgG antibodies to diphtheria toxoid (Dtx), tetanus toxoid (Ttx) and four pertussis antigens [pertussis toxoid (Ptx), fimbriae (Fim), filamentous haemagglutinin (FHA) and pertactin (Per)] was performed at the National Institute of Public Health and the Environment (RIVM), Netherlands using published protocols (35). Vaccine-induced IgG levels for Dtx and Ttx require a post-vaccination titer of ≥0.1 international units (IU)/mL for long-term protection, although lower levels of 0.01–0.1 IU/mL may provide some protection. There are no established protective levels for the pertussis antibodies.

### Whole Blood Cultures and Cytokine Multiplex Analysis

In addition to vaccine-specific cell mediated immune (CMI) responses, we also analyzed for reactivity to the unrelated antigen PPD and the general T cell stimulant anti-CD3/anti-CD28. 100μL aliquots of heparinized whole blood were cultured in 96-well U-bottom plates with a measles peptide-pool of 122 15mer peptides overlapping by 10 amino acids spanning the immunodominant measles haemagglutinin protein (all 1μg/mL final concentration, Sigma-Genosys, UK); tetanus toxoid (TT antigen) (10μg/ml, Sanofi Pasteur, France); purified protein derivative (PPD) (10μg/mL, Statens Serum Institute, Denmark); anti-CD3 (αCD3) (5μg/mL, Becton-Dickinson (BD), USA) and anti-CD28 (αCD28) (5μg/mL, eBiosciences, UK); and medium alone as a negative control. Plates were incubated for 16 h at 37°C, 5% CO_2_, centrifuged at 2,000 rpm for 5 min and supernatant collected and stored at −20°C.

Cytokine concentrations were analyzed in thawed culture supernatants using a customized 10-plex array [interleukin 1 beta [IL-1β], IL-4, interferon-gamma [IFN-γ], IL-10, IL-12(p70), eotaxin, granulocyte macrophage colony-stimulating factor [GM-CSF], platelet derived growth factor-BB [PDGF-BB], tumor necrosis factor [TNF], vascular endothelial growth factor [VEGF]] acquired using the Bio-Plex 200 Suspension Array system (Bio-Rad, Hercules, California, USA) as described previously (30). This panel was selected based on preliminary assays showing that these 10 cytokines/chemokines were differentially affected in the different vaccine groups. Pro-inflammatory to anti-inflammatory cytokine ratios were also analyzed to gain further insights into the effect of HCMV infection on the immune homeostatic response (36). Since TNF and IFN-γ are pivotal pro-inflammatory cytokines and IL-10 is a key anti-inflammatory cytokine we analyzed the IFN-γ:IL-4 ratio as an indication of the T helper 1 (Th1):Th2 balance and the TNF:IL-10 and IFN-y:IL-10 ratios. Assays were not performed for all infants due to insufficient blood volume, clotted or contaminated blood samples. Thus, 147 of the 225 infants included in the study had cytokine results (MV group *n* = 50, DTP group *n* = 55, MV+DTP group *n* = 42).

### Statistical Analysis

General linear modeling was used to estimate the geometric mean, geometric standard deviation (GSD), mean difference, 95% confidence interval and *p*-value for each variable for the different subject groups (infants with and without HCMV infection, different vaccine groups, males, and females). The effects of infant sex and vaccine groups were determined by estimating HCMV:sex interactions and HCMV:vaccine group interactions. Analysis was performed on natural logarithmic transformed antibody levels then converted back to natural numbers by exponential transformation; thus, the GSD is a multiplicative factor, and not an absolute value. Since univariate analysis is unlikely to capture the complex inter-relationships between the immune parameters tested, multivariate linear regression modeling was used. Infant sex, HCMV status and immune parameters (all other antibody results for antibody data and all other cytokine/chemokine values for CMI data) were considered potential covariates for adjustment. Those covariates selected by forward stepwise regression had an entry *p*-value of 0.15 and a removal *p*-value of 0.2 and were presented to the statistical model as z-scores of the natural logarithms of the raw values. Missing data were substituted by multiple imputation (using “mi estimate: glm” Stata syntax, with 50 imputations). Results are presented as unadjusted and adjusted for covariates, but only adjusted *p*-values are reported in the text unless otherwise stated. Fisher's exact tests were used to analyse the proportion of HCMV+ and HCMV– infants with antibody levels above and below the protective threshold. All statistical analyses were performed using Stata MP2 V16.0 (StataCorp, College Station, TX USA).

## Results

### Infant Characteristics

The infant characteristics at 9 months of age have been described elsewhere for this cohort of children (30, 31). HCMV status was determined for 225 infants (108 females and 117 males) at 9 months of age for whom we had paired blood samples at 10 months of age, among whom 153 (68%) were found to be HCMV+ and 72 (32%) were HCMV– (Table 1). An equivalent proportion of males and females were HCMV+: 77 (71.3%) of the 108 females and 76 (65%) of the 117 males (Chi-square = 1.04, *p* = 0.31). More than half (46/72, 63.9%) of the HCMV– infants at 9 months of age had acquired the infection by the 10 months of age, resulting in an 88% HCMV infection rate by this age.

### HCMV Infection Is Associated With Lower Tetanus IgG Responses Following DTP Vaccination

**Figure 1:**
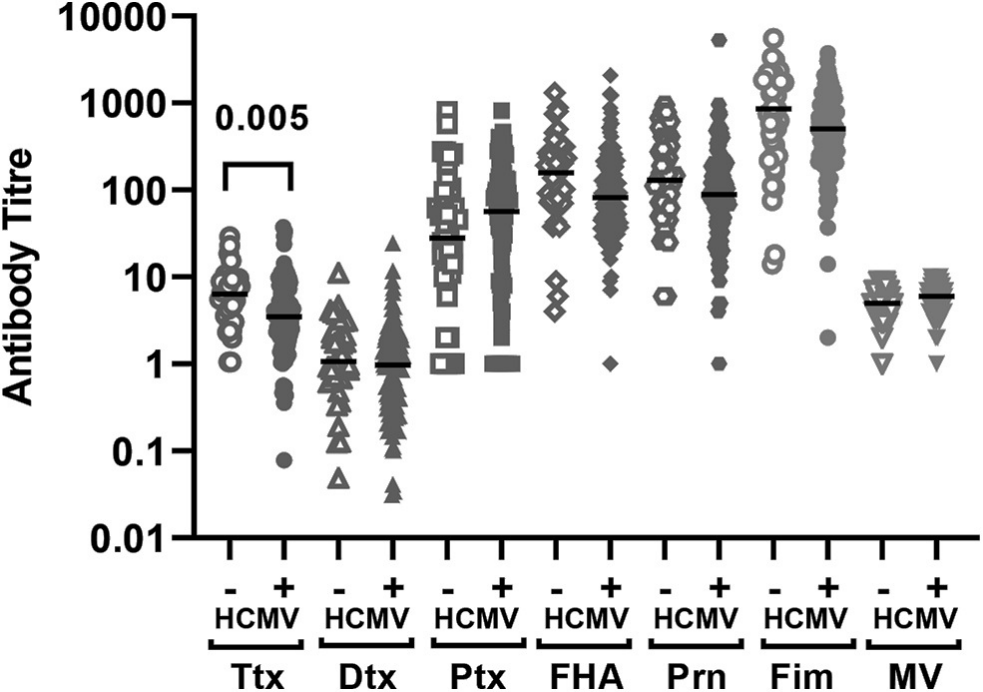
Vaccine antibodies 4 weeks after vaccination according to baseline HCMV status. Antibody titres to the vaccine antigens measured 4 weeks after vaccination (infants aged 10 months) according to HCMV status on the day of vaccination. The antibody titer values are shown on a logarithmic scale and each symbol represents an individual infant. The horizontal bar indicates the median value. Only significant adjusted *p*-values are shown in the figure indicating lower Ttx antibodies in HCMV+ as compared to HCMV- infants. Ttx, tetanus toxoid; Dtx, diphtheria toxoid; Ptx, pertussis toxoidl; FHA, filamaentous haemagglutinin; Prn, pertactin; Fim, fimbriae; MV, measles virus.

There was no significant difference in measles HAI geometric mean titres between the HCMV+ and HCMV- infants who received measles vaccination (*p* = 0.11) (Figure 1, Table 2). By contrast, the HCMV+ infants who received a DTP vaccination at 9 months of age (with or without MV) had lower tetanus toxoid IgG titres (*p* = 0.005) than the HCMV- individuals, while IgG responses to diphtheria toxoid and the 4 pertussis antigens (pertussis toxoid, pertactin, FHA, and fimbriae) did not appear to be significantly influenced by HCMV status (Figure 1, Table 2). Importantly, the presence of HCMV infection did not affect the proportion of donors achieving protective antibody levels to Ttx, Dtx or measles vaccination (Table 3) but this could not be analyzed for pertussis antigens for which protective levels have not been established. An analysis for the effect of sex similarly showed that Ttx IgG responses were significantly lower in DTP vaccinated HCMV+ males (*p* = 0.017), but the lower levels in HCMV+ females were only significant on unadjusted analysis (unadjusted *p* = 0.038, adjusted *p* = 0.13). Pertactin IgG responses were also lower in HCMV+ females compared to the uninfected females on unadjusted analysis (*p* = 0.033) but not after adjustment for covariables (*p* = 0.22), while IgG responses to all other vaccine antigens were comparable in males and females with or without HCMV (Supplementary Table 1).

**Table 2:** Effect of HCMV infection on vaccine antibody measurements.

	**HCMV–**		**HCMV+**		**Comparison^1^ (unadjusted)**			**Comparison^1^(adjusted) ^2^**		
	***N***	**Geo mean**	***N***	**Geo mean**	**Δ**	**95%CI**	***P*-value**	**Δ**	**95%CI**	***P*-value**
		**(GSD)**		**(GSD)**						
Ttx	39	5.73 (2.10)	104	3.43 (2.72)	−2.29	(−3.19 to −1.08)	**0.001**	−1.44	(−2.20 to −0.49)	**0.005**
Dtx	39	1.13 (3.02)	104	0.83 (3.40)	−0.30	(−0.59 to 0.12)	0.14	0.03	(−0.22 to 0.38)	0.82
Ptx	39	26.4 (6.4)	104	28.9 (7.5)	2.5	(−11.9 to 31.5)	0.80	10.9	(−5.1 to 43.0)	0.24
Fim	39	654 (4.0)	104	448 (3.0)	−206	(−376 to 66)	0.12	−86	(−235 to 134)	0.39
FHA	39	132 (3.5)	104	83 (3.2)	−49	(−79 to −2)	**0.044**	−14	(−39 to 22)	0.40
Prn	39	126.5 (3.5)	104	85.9 (3.6)	−40.6	(−72.2 to 9.4)	0.098	1.7	(−29.9 to 48.7)	0.93
MV	56	145 (8.9)	108	260 (9.1)	115	(−17 to 383)	0.11	147	(−21 to 507)	0.11

*Vaccine antibodies were measured 4 weeks after vaccination at 9 months of age. Ptx, Dtx, Ttx, Fim23, FHA and Prn were measured in the DTP and MV+DTP groups only, while MV was measured in the MV and MV+DTP groups only*.

1 *Estimated using general linear modeling. P < 0.05 are indicated in bold type*.

2 *Covariates selected for adjustment by forward stepwise regression. N, number of infants analyzed; Geo mean, geometric mean; GSD, geometric standard deviation; Δ, mean difference; 95% CI, 95% confidence interval; Dtx, diphtheria toxoid; Ttx, tetanus toxoid; Ptx, pertussis toxoid; FHA, filamaentous haemagglutinin; Prn, pertactin; Fim, fimbriae; MV, measles virus*.

**Table 3:** HCMV infection does not significantly affect protective antibody levels.

**Vaccine antibody**	**Protective**	**Below threshold (*****N*****)**		**Above threshold (*****N*****)**		**Fisher's exact test**
	**Threshold value**	**HCMV–**	**HCMV+**	**HCMV–**	**HCMV+**	***P*-value**
Dtx antibodies	≥0.1 IU/mL	1	3	38	101	1.0
Ttx antibodies	≥0.1 IU/mL	0	1	39	103	1.0
Measles antibodies	≥3 log2 titer	5	9	51	99	1.0

*Number of infants above and below the post-vaccination protective threshold antibody levels according to HCMV status. N, number of infants; Dtx, diphtheria toxoid; Ttx, tetanus toxoid*.

Analysis was also performed separately for the individual vaccine groups to see if the dual administration of MV with DTP influenced the effect of HCMV for each vaccine antigen (Supplementary Table 1). This showed that the HCMV+ infants in the MV+DTP group had lower Ttx titres (*p* = 0.012) compared to HCMV– infants, whereas the effect was only significant in the DTP alone group on unadjusted but not adjusted analysis (*p* = 0.19). While not the case for the DTP and MV+DTP infants combined, Dtx IgG levels were lower in HCMV+ infants who received DTP alone on unadjusted analysis (< 0.001) but not after adjusting for other variables (*p* = 0.22). The same applied to unadjusted analysis of IgG responses to Fim, FHA and Pertactin which were significantly lower in HCMV+ individuals in the DTP (*p* = 0.031, *p* = 0.05, *p* = 0.003, respectively) but not those that received DTP+MV, and not significant after adjusting for other variables (Supplementary Table 1).

### Suppressed Overall T Cell and PPD Reactivity in HCMV Infected Infants

Stimulation of infant whole blood with anti-CD3 plus anti-CD28 was performed to analyse overall non-antigen specific T cell reactivity at baseline (Table 4). Univariate analysis showed a significantly lower IL-4 (*p* = 0.002), TNF (*p* = 0.012), eotaxin (*p* = 0.02), and TNF:IL-10 ratio (*p* < 0.0001) in culture supernatants from HCMV infected as compared to HCMV uninfected infants, but only IL-4 was significant on adjusting for covariates (*p* = 0.046). Responses to the mycobacterial antigen purified protein derivative (PPD) were also generally suppressed in those infected with HCMV as evidenced by lower unadjusted PPD-stimulated IL-4 (*p* = 0.025), IL-1β (*p* = 0.039), eotaxin (*p* = 0.003), GMCSF (*p* = 0.036), TNF (*p* = 0.021), and TNF:IL-10 ratio (*p* = 0.004) in the HCMV+ compared to the HCMV– group, of which only GMCSF (*p* = 0.021) was significant after multivariate adjustment, and IL-12(p70) (*p* = 0.035) and IL-10 (*p* = 0.044) were also significant after adjusting (Table 4). Of note, the unstimulated cytokine levels in whole blood supernatants were unaffected by HCMV status (Supplementary Table 2).

**Table 4:** Effect of HCMV infection on cytokine responses to T cell stimulation with aCD3/CD28 at 9 months of age.

	**HCMV–**		**HCMV+**		**Comparison^1^ (unadjusted)**			**Comparison^1^ (adjusted) ^2^**		
	***N***	**Geo mean**	**N**	**Geo mean**	**Δ**	**95%CI**	***P*-value**	**Δ**	**95%CI**	***P*-value**
		**(GSD)**		**(GSD)**						
IL-4	48	18.7 (2.5)	98	10.2 (4.2)	−8.6	(−11.8 to −3.9)	**0.002**	−2.5	(−4.4 to −0.1)	**0.046**
IL-1β	48	2584 (11)	98	1204 (15)	−1380	(−2072 to 250)	0.080	−27	(−452 to 561)	0.92
IL-10	48	1481 (8.1)	98	1090 (8.4)	−391	(−952 to 763)	0.41	299	(−19 to 719)	0.068
IL-12(p70)	48	99 (2.7)	98	99 (3.5)	0	(−31 to 45)	0.99	8	(−10 to 29)	0.41
Eotaxin	48	268 (2.9)	98	169 (3.5)	−99	(−153 to −19)	**0.020**	−7	(−47 to 43)	0.76
GMCSF	48	23.6 (12.7)	98	14.8 (11.5)	−8.9	(−17.4 to 11.3)	0.28	−5.8	(−14.2 to 12.5)	0.42
IFN-γ	48	2810 (5.8)	98	1939 (8.2)	−871	(−1794 to 889)	0.26	185	(−559 to 1296)	0.68
PDGFBB	48	8617 (2.5)	98	8776 (2.0)	159	(−2042 to 3097)	0.90	520	(−1378 to 2932)	0.62
TNF	48	4402 (16.3)	98	1162 (32.1)	−3240	(−3992 to −1107)	**0.012**	−565	(−1109 to 251)	0.15
VEGF	48	639 (2.6)	98	607 (4.1)	−32	(−228 to 257)	0.80	−35	(−135 to 86)	0.55
TNF/IL-10	48	2.97 (4.25)	98	1.07 (6.68)	−1.91	(−2.36 to −1.12)	** < 0.001**	−0.53	(−1.00 to 0.20)	0.13
IFN-γ/IL-10	48	3.95 (4.52)	98	4.80 (4.67)	0.85	(−1.10 to 4.14)	0.46	0.78	(−1.17 to 4.07)	0.51
IFN-γ/IL-4	48	150 (4.8)	98	191 (4.1)	41	(−37 to 171)	0.37	30	(−34 to 125)	0.41
**Analysis by Sex**										
**IL-4**										
Male	24	20.1 (2.3)	47	9.6 (4.9)	−10.4	(−14.5 to −3.3)	**0.009**	−3.7	(−6.4 to −0.3)	**0.035**
Female	24	17.5 (2.7)	51	10.7 (3.7)	−6.8	(−11.2 to 0.6)	0.067	−1.2	(−4.2 to 2.7)	0.51
**Eotaxin**										
Male	24	256 (3.2)	47	194 (4.3)	−61	(−151 to 106)	0.39	49	(−16 to 141)	0.16
Female	24	281 (2.5)	51	149 (2.9)	−132	(−188 to −44)	**0.007**	−59	(−103 to 0)	**0.049**
**TNF**										
Male	24	4624 (18.8)	47	809 (35.0)	−3816	(−4450 to −881)	**0.026**	−1333	(−1862 to −393)	**0.011**
Female	24	4189 (15.0)	51	1622 (29.7)	−2567	(−3793 to 2453)	0.187	274	(−633 to 1839)	0.63
TNF/IL-10										**0.046**
Male	24	2.89 (4.37)	47	0.85 (7.88)	−2.04	(−2.52 to −0.95)	**0.004**	−0.94	(−1.43 to −0.04)	**0.043**
Female	24	3.06 (4.27)	51	1.31 (5.62)	−1.75	(−2.43 to −0.31)	**0.025**	−0.07	(−0.84 to 1.36)	0.90

*Whole blood was cultured with aCD3/aCD28 and cytokines measured by multiplex assay in the culture supernatants after 18 h of culture*.

1 *Estimated using general linear modeling. P < 0.05 are indicated in bold type*.

2 *Covariates selected for adjustment by forward stepwise regression as described in methods. Only those cytokines which showed significant effects of HCMV infection in the sex analysis are shown. N, number of infants analyzed; Geo mean, geometric mean; GSD, geometric standard deviation; Δ, mean difference; 95% CI, 95% confidence interval*.

When analyzing separately by sex the lower reactivity in the HCMV+ infants compared to the HCMV- group was also apparent but only significant in one sex or the other. Thus, HCMV+ males but not females had significantly lower aCD3aCD28 stimulated IL-4 (*p* = 0.035), TNF (*p* = 0.011), and TNF:IL-10 (*p* = 0.043); while HCMV+ females but not males had lower aCD3aCD28 stimulated eotaxin (*p* = 0.049) than their HCMV– counterparts (Table 4). PPD reactivity showed a similar pattern with HCMV+ males having lower PPD-stimulated TNF:IL-10 ratios (*p* = 0.033); and females but not males having lower PPD-stimulated GMCSF (*p* = 0.003) (Table 5).

**Table 5:** Effect of HCMV infection on cytokine responses to PPD at 9 months of age.

	**HCMV–**		**HCMV+**		**Comparison^1^ (unadjusted)**			**Comparison^1^ (adjusted) ^2^**		
	***N***	**Geo mean**	***N***	**Geo mean**	**Δ**	**95%CI**	***P*-value**	**Δ**	**95%CI**	***P*-value**
		**(GSD)**		**(GSD)**						
IL-4	48	12.2 (4.1)	98	7.0 (4.0)	−5.2	(−7.9 to −0.8)	**0.025**	−1.8	(−4.3 to 2.0)	0.31
IL-1β	48	1245 (11)	98	515 (11)	−730	(−1022 to −55)	**0.039**	4	(−195 to 282)	0.98
IL-10	48	739 (6.5)	98	569 (5.8)	−171	(−437 to 329)	0.41	186	(4 to 432)	**0.044**
IL-12(p70)	48	76.3 (3.2)	98	74.1 (4.7)	−2.2	(−28.9 to 39.7)	0.90	16.1	(1.0 to 34.6)	**0.035**
Eotaxin	48	229 (3.1)	98	117 (4.8)	−113	(−155 to −47)	**0.003**	−50	(−97 to 23)	0.15
GMCSF	48	21.7 (13.0)	98	8.5 (12.4)	−13.2	(−18.2 to −1.3)	**0.036**	−13.8	(−18.5 to −3.0)	**0.021**
IFN-γ	48	2951 (5.5)	98	2730 (5.1)	−221	(−1417 to 1907)	0.79	1023	(−78 to 2705)	0.073
PDGFBB	48	7417 (2.5)	98	6060 (2.2)	−1357	(−2953 to 811)	0.20	−352	(−1617 to 1227)	0.63
TNF	48	1336 (18)	98	409 (19)	−926	(−1186 to −219)	**0.021**	−144	(−342 to 163)	0.30
VEGF	48	523 (3.0)	98	429 (3.5)	−94	(−234 to 115)	0.33	−69	(−140 to 17)	0.11
TNF/IL-10	48	1.81 (6.19)	98	0.72 (6.62)	−1.09	(−1.42 to −0.45)	**0.004**	−0.37	(−0.71 to 0.18)	0.16
IFN-γ/IL-10	48	3.95 (4.52)	98	4.80 (4.67)	0.85	(−1.10 to 4.14)	0.46	0.78	(−1.17 to 4.07)	0.51
IFN-γ/IL-4	48	241 (5.6)	98	388 (4.0)	148	(−18 to 436)	0.091	165	(−9 to 479)	0.069
**Analysis by Sex**										
GMCSF										
Male	24	16.0 (14.3)	47	7.8 (13.6)	−8.2	(−13.9 to 12.2)	0.27	−7.7	(−12.1 to 7.4)	0.20
Female	24	29.5 (12.0)	51	9.2 (11.6)	−20.3	(−26.7 to 0.5)	0.053	−23.6	(−30.9 to −3.5)	**0.030**
**TNF/IL-10**										
Male	24	1.92 (6.07)	47	0.67 (7.25)	−1.25	(−1.65 to −0.27)	**0.023**	−0.63	(−0.93 to −0.07)	**0.033**
Female	24	1.69 (6.55)	51	0.77 (6.16)	−0.93	(−1.38 to 0.17)	0.081	−0.02	(−0.62 to 1.24)	0.97

*Whole blood was cultured with PPD and cytokines measured by multiplex assay in the culture supernatants after 18 h of culture*.

1 *Estimated using general linear modeling. P < 0.05 are indicated in bold type*.

2 *Covariates selected for adjustment by forward stepwise regression as described in methods. Only those cytokines which showed significant effects of HCMV infection in the sex analysis are shown. N, number of infants analyzed; Geo mean, geometric mean; GSD, geometric standard deviation; Δ, mean difference; 95% CI, 95% confidence interval*.

### Minimal Effect of HCMV Infection on Vaccine-Specific Cellular Reactivity to Vaccination

Whole blood collected 4 weeks after vaccination was stimulated overnight with a measles peptide pool to determine measles-specific cellular reactivity in infants who received a measles vaccine (MV and MV+DTP groups), and responses to tetanus toxoid (TT antigen) were analyzed in infants who received a DTP vaccine (DTP and MV+DTP groups). Analysis for the impact of HCMV infection on the day of vaccination on the subsequent *in vitro* cytokine responses to the vaccine antigens showed very little consequence of HCMV infection on responses to measles vaccination, with only VEGF responses being lower (*p* = 0.002) in the HCMV+ compared to HCMV- infants (Table 6). Analysis separately in males and females showed that HCMV+ males had higher measles-specific IL-12(p70) (*p* = 0.024) and lower VEGF (*p* = 0.003) than the HCMV– males; while HCMV+ females had higher measles-specific GMCSF than their uninfected counterparts (*p* = 0.04) (Table 6). When the MV and MV+DTP groups were analyzed separately the measles stimulated eotaxin (*p* = 0.017) and VEGF (*p* = 0.016) were both lower in the HCMV+ MV+DTP group, but not those who received MV alone (Supplementary Table 3).

**Table 6:** Effect of baseline HCMV status on measles-specific cytokine responses 4 weeks after vaccination.

	**CMV–**		**CMV+**		**Comparison^1^ (unadjusted)**			**Comparison^1^ (adjusted) ^2^**		
	***N***	**Geo mean**	***N***	**Geo mean**	**Δ**	**95%CI**	***P*-value**	**Δ**	**95%CI**	***P*-value**
		**(GSD)**		**(GSD)**						
IL-4	33	4.72 (4.80)	72	5.91 (4.82)	1.19	(−1.58 to 6.41)	0.49	−0.31	(−1.60 to 1.49)	0.70
IL-1β	33	274 (24.1)	72	389 (28.8)	115	(−169 to 1163)	0.60	22	(−64 to 160)	0.67
IL-10	33	141 (14)	72	175 (9)	33	(−77 to 334)	0.68	−15	(−50 to 38)	0.53
IL-12(p70)	33	43.3 (6.4)	72	56.5 (4.4)	13.3	(−15.2 to 70.9)	0.46	21.8	(0.1 to 54.6)	**0.049**
Eotaxin	33	183 (5.9)	72	259 (5.0)	76	(−54 to 335)	0.33	−8	(−84 to 113)	0.87
GMCSF	33	2.38 (21.7)	72	5.17 (17.9)	2.79	(−0.85 to 15.1)	0.21	1.00	(−0.46 to 4.09)	0.24
IFN-γ	33	1411 (4.8)	72	1862 (6.3)	451	(−456 to 2219)	0.42	−144	(−587 to 521)	0.62
PDGFBB	33	5199 (2.7)	72	6819 (2.8)	1620	(−637 to 4994)	0.19	1743	(−207 to 4489)	0.086
TNF	33	174 (27)	72	306 (24)	132	(−91 to 960)	0.40	27	(−43 to 144)	0.52
VEGF	33	373 (4.9)	72	220 (9.8)	−153	(−268 to 87)	0.16	−210	(−282 to −94)	**0.002**
TNF/IL-10	33	1.23 (4.35)	72	1.71 (5.77)	0.48	(−0.32 to 1.99)	0.30	0.14	(−0.43 to 1.12)	0.69
IFN-γ/IL-10	33	9.98 (6.06)	72	10.5 (5.54)	0.55	(−4.82 to 11.5)	0.88	2.07	(−3.58 to 12.4)	0.56
IFN-γ/IL-4	33	299 (3.5)	72	317 (4.2)	18	(−111 to 237)	0.82	−9	(−129 to 191)	0.91
**Analysis by Sex**										
**IL-12(p70)**										
Male	17	41.6 (5.1)	31	51.3 (4.7)	9.7	(−20.8 to 84.8)	0.65	34.6	(3.3 to 92.1)	**0.024**
Female	16	45.2 (8.5)	41	61.2 (4.2)	16.0	(−25.0 to 140)	0.59	7.3	(−17.3 to 48.6)	0.63
**GMCSF**										
Male	17	5.52 (31.0)	31	3.55 (15.0)	−1.97	(−4.93 to 16.0)	0.63	−0.43	(−1.79 to 3.68)	0.74
Female	16	0.93 (10.8)	41	7.00 (20.4)	6.07	(0.70 to 29.2)	**0.007**	2.33	(0.06 to 8.71)	**0.040**
**VEGF**										
Male	17	476 (3.7)	31	156 (14.0)	−320	(−423 to −22)	**0.041**	−302	(−379 to −143)	**0.003**
Female	16	283 (6.4)	41	290 (6.9)	6	(−183 to 553)	0.97	−119	(−223 to 69)	0.17

*Whole blood was cultured with a measles peptide pool (results for measles vaccinated infants only shown) and cytokines measured by multiplex assay in the culture supernatants after 18 h of culture*.

1 *Estimated using general linear modeling. P < 0.05 are indicated in bold type*.

2 *Covariates selected for adjustment by forward stepwise regression as described in methods. Only those cytokines which showed significant effects of HCMV infection in the sex analysis are shown. N, number of infants analyzed; Geo mean, geometric mean; GSD, geometric standard deviation; Δ, mean difference; 95% CI, 95% confidence interval*.

Among the infants who received a DTP vaccine, there was a significantly lower TT-stimulated GMCF (*p* = 0.009) and eotaxin (*P* = 0.001) response among those infected with HCMV compared to the uninfected cohort (Table 6). Analysis in the individual sexes showed that HCMV infected females had higher IL-1β (*p* = 0.025), TNF (*p* = 0.014), and PDGFBB (*p* = 0.05) in TT cultures whereas males had lower GMCSF (*p* = 0.013) (Table 7). Analysis of the MV+DTP and DTP groups separately showed that the MV+DTP group had significantly lower IL-1β (*p* = 0.03), and higher eotaxin (*p* = 0.035) and TNF responses to TT (*p* = 0.005) among HCMV infected infants while the DTP alone group showed no significant effect of HCMV infection (Supplementary Table 3).

**Table 7:** Effect of baseline HCMV status on TT-specific cytokine responses 4 weeks after vaccination.

	**CMV–**		**CMV+**		**Comparison^1^ (unadjusted)**			**Comparison^1^ (adjusted) ^2^**		
	***N***	**Geo mean**	***N***	**Geo mean**	**Δ**	**95%CI**	***P*-value**	**Δ**	**95%CI**	***P*-value**
		**(GSD)**		**(GSD)**						
IL-4	28	6.66 (3.48)	68	7.86 (4.13)	1.21	(−2.16 to 7.10)	0.56	0.09	(−1.16 to 1.72)	0.90
IL-1β	28	750 (22)	68	716 (23)	−34	(−560 to 1947)	0.95	−99	(−215 to 141)	0.32
IL-10	28	202 (10)	68	405 (12)	204	(−55 to 917)	0.18	30	(−36 to 134)	0.44
IL-12(p70)	28	32.2 (5.8)	68	50.1 (5.3)	17.9	(−8.4 to 73.4)	0.24	11.8	(−9.3 to 46.2)	0.33
Eotaxin	28	90 (5.3)	68	202 (4.9)	112	(9 to 319)	**0.026**	53	(−5 to 149)	0.078
GMCSF	28	44.5 (12.1)	68	24.8 (22.5)	−19.6	(−36.7 to 34.7)	0.33	−11.9	(−17.3 to −0.9)	**0.038**
IFN-γ	28	1259 (4.6)	68	1603 (7.1)	344	(−476 to 2023)	0.51	−296	(−655 to 274)	0.26
PDGFBB	28	9002 (2.9)	68	10096 (4.8)	1094	(−3065 to 8166)	0.67	52	(−3149 to 5126)	0.98
TNF	28	352 (11)	68	781 (21)	429	(−99 to 2066)	0.17	137	(−5 to 403)	0.063
VEGF	28	216 (8.0)	68	246 (8.4)	31	(−116 to 390)	0.77	−19	(−101 to 115)	0.73
TNF/IL-10	28	1.75 (4.91)	68	1.90 (4.16)	0.15	(−0.77 to 1.93)	0.81	0.24	(−0.54 to 1.77)	0.63
IFN-γ/IL-10	28	6.24 (5.88)	68	3.94 (4.60)	−2.30	(−4.34 to 1.93)	0.22	−1.98	(−4.34 to 2.25)	0.29
IFN-γ/IL-4	28	199 (3.6)	68	204 (3.2)	5	(−80 to 149)	0.93	10	(−73 to 154)	0.86
**Analysis by Sex**										
**IL-1β**										
Male	11	652 (28)	33	516 (36)	−136	(−594 to 3919)	0.83	145	(−100 to 892)	0.37
Female	17	827 (19)	35	984 (14)	157	(−632 to 4122)	0.83	−225	(−303 to −42)	**0.025**
**GMCSF**										
Male	11	48.7 (16.6)	33	11.8 (25.4)	−36.9	(−46.9 to 28.6)	0.14	−23.1	(−28.4 to −7.9)	**0.013**
Female	17	41.7 (9.5)	35	51.1 (17.4)	9.4	(−29.1 to 166)	0.78	−3.5	(−12.3 to 18.5)	0.64
**PDGFBB**										
Male	11	12605 (2.4)	33	6252 (7.2)	−6353	(−9853 to 1597)	0.094	−5892	(−9785 to 2420)	0.13
Female	17	7098 (3.0)	35	16081 (2.2)	8983	(1906 to 21624)	**0.006**	3871	(2 to 10097)	**0.050**
**TNF**										
Male	11	365 (10)	33	358 (27)	−7	(−298 to 1552)	0.98	44	(−103 to 459)	0.68
Female	17	343 (13)	35	1667 (14)	1324	(40 to 6899)	**0.035**	234	(32 to 639)	**0.014**

*Whole blood was cultured with tetanus toxoid for those infants vaccinated with DTP (DTP and MV+DTP groups) and cytokines measured by multiplex assay in the culture supernatants after 18 h of culture*.

1 *Estimated using general linear modeling. P < 0.05 are indicated in bold type*.

2 *Covariates selected for adjustment by forward stepwise regression as described in methods. Only those cytokines which showed significant effects of HCMV infection in the sex analysis are shown. N, number of infants analyzed; Geo mean, geometric mean; GSD, geometric standard deviation; Δ, mean difference; 95% CI, 95% confidence interval*.

## Conclusions

As expected, a high incidence of HCMV infection was found in this Gambian infant cohort, reaching 88% by 10 months of age (5). Previous studies of the impact of HCMV infection on vaccine responses have variously described decreased, unaffected and enhanced antibody responses in the elderly; while the handful of early life studies have shown no effect on antibody responses to measles, rubella, meningococcus A and C, tetanus and Hib (25). The present study largely suggests minimal effects of HCMV infection on antibody responses to vaccination with diphtheria-tetanus-whole cell pertussis vaccine or measles vaccine at 9 months of age. Indeed, only Ttx titres were convincingly lower in infants infected with HCMV compared to the uninfected infants, while measles HAI titres and responses to four pertussis antigens and diphtheria toxoid were not affected by HCMV status. Analysis in the individual sexes confirmed significantly lower Ttx IgG levels in HCMV+ males but the lower levels in HCMV+ females were not significantly different to HCMV– females, probably due to lower numbers when analyzing the sexes separately. Thus, there was little evidence for sex differential effects of HCMV infection on antibody responses in our study. These lower Ttx titres are unlikely to be of major significance since HCMV+ infants still generally had levels well above the protective threshold. There is a suggestion that vaccination with DTP alone led to lower levels to other vaccine antigens—Dtx, Fim, FHA and Pertactin—in the HCMV+ infants but not when MV was given with DTP suggesting a boosting effect of MV co-administration, but this observation was only significant on unadjusted analysis. The live vaccine bacille Calmette-Guérin has similarly been shown to boost antibody responses to co-administered vaccines (37, 38).

HCMV infection is known to lead to expansion of terminally differentiated T cells, particularly in the CD8 T cell subset (5). One might therefore predict that this would impact cellular responses to vaccination, although this has not been investigated except for the one study showing an early decline in measles-specific CD4 T cell IFN-γ responses in HCMV infected infants (8). We found little evidence for a major impact of HMCV infection on vaccine-induced cellular immunity to measles or DTP vaccination. This is interesting because HCMV infection seemed to lead to lower pro-inflammatory (TNF, IL1β, eotaxin, GMCSF) and Th2 (IL-4) reactivity to general T cell stimulation and the mycobacterial antigen PPD as compared to the HCMV- infants. The lower responses in HCMV+ infants were apparent in both males and females but involved different cytokine profiles in the sexes: TNF and the Th2 cytokine IL-4 for males and the innate cytokines eotaxin and GMCSF for females. When analyzing measles-specific immunity, the only cytokine response apparently affected by HCMV infection was the growth factor VEGF, which was lower in HCMV+ infants. The lack of impact of HCMV infection on measles-specific innate pro-inflammatory cytokines IL-1β and TNF, the Th1 cytokine IFN-γ, the Th2 cytokine IL-4 and the anti-inflammatory cytokine IL-10 is encouraging. The clinical significance of lower VEGF production is uncertain but it is an important vascular growth factor that has also been linked with trauma and malignancy (39, 40). Effects on tetanus toxoid-specific responses similarly showed lower GMCSF and eotaxin in the HCMV+ infants but no effect on innate pro-inflammatory, Th1, Th2 cytokines or IL-10.

HCMV+ males exhibited higher measles-specific IL-12(p70) production, while females had higher measles-specific GMCF and higher TT-cultured pro-inflammatory innate cytokines (IL-1β and TNF) and PDGFBB as compared to their HCMV- counterparts. This suggests that HCMV infection may have a greater enhancing effect on vaccine-specific cell mediated immunity in females, with mixed effects among males. The finding of sex differences in HCMV effects are consistent with the recent studies showing sex differences in immunological responses to vaccination (28, 29). More robust immune responses in females as compared to males have been reported in the vaccine literature, but sex differences in the immunological consequences of HCMV infection have not previously been described. Differences in sex hormone levels in infant males and females and the multiple X-linked immune response genes have been implicated mechanistically in sex-differential immunity but have yet to be confirmed as the cause. Sex differences in the gut microbiota may also play a role (41) given the studies suggesting that the microbiota influences vaccine-specific immunity (42). Furthermore, non-human primate studies suggest that latent HCMV infection alters the gut microbiota composition, which the authors propose could lead to heterogeneity in responses to vaccines (43), although this has not been studied in humans.

Several limitations of this study should be borne in mind. One is that some infants who acquired HCMV early in life may no longer be shedding virus in their urine by 9 months of age. However, previous longitudinal studies of HCMV carriage from birth suggest that viral shedding is quite persistent in early life (44) and that only a minority of those infected earlier will test negative at 9 months of age (5). Also, many of those infants who were HCMV negative at 9 months of age had become positive by 10 months of age when the vaccine responses were measured. Since it is not possible to determine at what point they became positive we have to accept that HCMV acquisition shortly after vaccination could also have had an effect on responses to vaccination and interfered with our results.

We therefore find little evidence that the very high levels of HCMV infection in African infants has a major impact of vaccine-specific immunity despite the profound polyclonal expansion and terminal differentiation of their T cells. This is in keeping with the existing limited studies but would suggest that almost universal HCMV infection by 1 year of age in many low-income countries does not prevent them responding adequately to vaccination. From an evolutionary point of view this is a good strategy for this highly successful virus that is able to establish life-long latent infection from an early age. Indeed, the opinion is now swaying to consider HCMV infection beneficial to the immune system in younger years, whilst becoming detrimental in older individuals (45). This study, in combination with those already published, should therefore alleviate any concerns that early HCMV infection significantly diminishes vaccine induced protective immunity, even when consideration of sex is taken into account.

## Data Availability Statement

The raw data supporting this paper will be made available by the authors upon request.

## Ethics Statement

The studies involving human participants were reviewed and approved by Joint Gambia Government/MRC Ethics Committee. Written informed consent to participate in this study was provided by the participants' legal guardian/next of kin.

## Author Contributions

KF, HW, and SR-J conceived and designed the study. JA recruited the study participants. MC, FN-K, JN-J, LS, and AD performed the laboratory assays. IR performed the statistical analysis. KF and MC wrote the first draft of the manuscript. All authors contributed to the manuscript revision, read, and approved the submitted version.

## Conflict of Interest

The authors declare that the research was conducted in the absence of any commercial or financial relationships that could be construed as a potential conflict of interest.
