# Supplementary material for: Limited Impact of Human Cytomegalovirus Infection in African Infants on Vaccine-Specific Responses Following Diphtheria-Tetanus-Pertussis and Measles Vaccination
Source: Front Immunol. 2020 Jun 5;11:1083. doi: 10.3389/fimmu.2020.01083 (PMC7291605; doi:10.3389/fimmu.2020.01083)
Supplement: Supplementary file 1 [file Table_1.DOCX]

Supplementary Material

**Supplementary Table 1 Effect of CMV infection on vaccine antibody measurements by sex and vaccination group**

Vaccine antibodies 4 weeks after vaccination analysed by infant sex and vaccine group. Ptx, Dtx, Ttx, Fim23, FHA and Prn were measured in the DTP and MV+DTP groups only, while MV was measured in the MV and MV+DTP groups only. ^1^Estimated using general linear modelling. P-values <0.05 are indicated in bold type. ^2^Covariates selected for adjustment by forward stepwise regression as described in methods. N = number of infants analysed, Geo mean = geometric mean, GSD = geometric standard deviation, Δ = mean difference, 95% CI = 95% confidence interval, Dtx = diphtheria toxoid, Ttx = tetanus toxoid, Ptx = pertussis toxoid, FHA = filamaentous haemagglutinin, Prn = pertactin, Fim = fimbriae, MV = measles virus.

|  | **CMV-** | | | **CMV+** | | | | **Comparison^1^ (unadjusted)** | | | | | | | | | **Comparison^1^ (adjusted) ^2^** | | | | | | | | |  |
| --- | --- | --- | --- | --- | --- | --- | --- | --- | --- | --- | --- | --- | --- | --- | --- | --- | --- | --- | --- | --- | --- | --- | --- | --- | --- | --- |
|  | | **N** | **Geo Mean (GSD)** | | **N** | | **Geo Mean (GSD)** | | **Δ** | | | **95%CI** | | | **P-value** | | | **Δ** | | | **95%CI** | | | **P-value** | | |
| Pertussis toxin antibodies (Ptx) | | | |  | |  | |  | |  | | |  | | |  | | |  | | | |  | |  |  |
| Male | 19 | | 15.9 (6.8) | 52 | | | 17.4 (8.2) | 1.6 | | | (-9.5 to 32.1) | | | 0.86 | | | 6.2 | | | (-6.2 to 41.2) | | 0.46 | | | |  |
| Female | 20 | | 41.8 (5.5) | 52 | | | 47.5 (6.1) | 5.8 | | | (-22.2 to 73.7) | | | 0.77 | | | 19.8 | | | (-10.9 to 93.1) | | 0.29 | | | |  |
| MV+DTP | 25 | | 26.8 (7.2) | 65 | | | 34.9 (7.2) | 8.1 | | | (-12.6 to 58.7) | | | 0.56 | | | 16.1 | | | (-6.8 to 71.1) | | 0.24 | | | |  |
| DTP | 14 | | 25.5 (5.4) | 39 | | | 21.0 (8.0) | -4.5 | | | (-18.3 to 35.8) | | | 0.72 | | | 5.2 | | | (-10.1 to 51.9) | | 0.65 | | | |  |
| Diphtheria toxin antibodies (Dtx) | | | |  | | |  |  | | |  | | |  | | |  | | |  | |  | | | |  |
| Male | 19 | | 1.32 (2.47) | 52 | | | 0.79 (3.55) | -0.54 | | | (-0.86 to 0.00) | | | 0.051 | | | -0.21 | | | (-0.49 to 0.21) | | 0.28 | | | |  |
| Female | 20 | | 0.98 (3.58) | 52 | | | 0.87 (3.28) | -0.11 | | | (-0.52 to 0.65) | | | 0.71 | | | 0.24 | | | (-0.13 to 0.86) | | 0.25 | | | |  |
| MV+DTP | 25 | | 0.89 (3.51) | 65 | | | 0.86 (3.53) | -0.03 | | | (-0.40 to 0.63) | | | 0.92 | | | 0.21 | | | (-0.12 to 0.69) | | 0.25 | | | |  |
| DTP | 14 | | 1.78 (1.78) | 39 | | | 0.77 (3.22) | -1.00 | | | (-1.29 to -0.55) | | | **<0.001** | | | -0.28 | | | (-0.58 to 0.22) | | 0.22 | | | |  |
| Tetanus toxin antibodies (Ttx) | | | |  | | |  |  | | |  | | |  | | |  | | |  | |  | | | |  |
| Male | 19 | | 5.64 (1.56) | 52 | | | 3.35 (3.00) | -2.29 | | | (-3.29 to -0.86) | | | **0.004** | | | -1.55 | | | (-2.46 to -0.33) | | **0.017** | | | |  |
| Female | 20 | | 5.81 (2.59) | 52 | | | 3.52 (2.45) | -2.29 | | | (-3.62 to -0.16) | | | **0.038** | | | -1.33 | | | (-2.54 to 0.48) | | 0.13 | | | |  |
| MV+DTP | 25 | | 4.72 (2.03) | 65 | | | 3.10 (3.06) | -1.62 | | | (-2.62 to -0.14) | | | **0.034** | | | -1.49 | | | (-2.32 to -0.38) | | **0.012** | | | |  |
| DTP | 14 | | 8.20 (1.98) | 39 | | | 4.07 (2.09) | -4.12 | | | (-5.50 to -2.03) | | | **0.001** | | | -1.39 | | | (-2.87 to 0.83) | | 0.19 | | | |  |
| Fimbriae |  | |  |  | | |  |  | | |  | | |  | | |  | | |  | |  | | | |  |
| Male | 19 | | 579 (4.1) | 52 | | | 384 (2.7) | -195 | | | (-384 to 177) | | | 0.24 | | | -119 | | | (-283 to 164) | | 0.34 | | | |  |
| Female | 20 | | 730 (4.0) | 52 | | | 520 (3.3) | -210 | | | (-463 to 281) | | | 0.32 | | | -35 | | | (-254 to 320) | | 0.81 | | | |  |
| MV+DTP | 25 | | 502 (4.5) | 65 | | | 402 (2.8) | -100 | | | (-287 to 249) | | | 0.49 | | | -39 | | | (-198 to 214) | | 0.72 | | | |  |
| DTP | 14 | | 1068 (2.7) | 39 | | | 538 (3.3) | -531 | | | (-781 to -65) | | | **0.031** | | | -230 | | | (-466 to 188) | | 0.22 | | | |  |
| FHA |  | |  |  | | |  |  | | |  | | |  | | |  | | |  | |  | | | |  |
| Male | 19 | | 127 (3.3) | 52 | | | 88 (4.1) | -40 | | | (-81 to 40) | | | 0.26 | | | -8 | | | (-49 to 60) | | 0.78 | | | |  |
| Female | 20 | | 136 (3.9) | 52 | | | 78 (2.5) | -58 | | | (-94 to 10) | | | 0.084 | | | -19 | | | (-46 to 22) | | 0.31 | | | |  |
| MV+DTP | 25 | | 119 (4.2) | 65 | | | 78 (3.5) | -40 | | | (-77 to 27) | | | 0.19 | | | -29 | | | (-58 to 15) | | 0.17 | | | |  |
| DTP | 14 | | 160 (2.5) | 39 | | | 91 (2.8) | -69 | | | (-108 to 0) | | | **0.050** | | | 13 | | | (-27 to 81) | | 0.59 | | | |  |
| Pertactin |  | |  |  | | |  |  | | |  | | |  | | |  | | |  | |  | | | |  |
| Male | 19 | | 93.8 (2.5) | 52 | | | 94.2 (4.3) | 0.4 | | | (-40.0 to 71.2) | | | 0.99 | | | 33.9 | | | (-12.3 to 115) | | 0.19 | | | |  |
| Female | 20 | | 165.8 (4.3) | 52 | | | 78.4 (2.9) | -87.4 | | | (-126 to -9.9) | | | **0.033** | | | -32.7 | | | (-67.4 to 25.3) | | 0.22 | | | |  |
| MV+DTP | 25 | | 98.3 (3.8) | 65 | | | 87.1 (4.1) | -11.2 | | | (-51.3 to 63.0) | | | 0.70 | | | 20.1 | | | (-21.1 to 88.1) | | 0.41 | | | |  |
| DTP | 14 | | 201.9 (2.5) | 39 | | | 83.8 (2.8) | -118 | | | (-155 to -53.4) | | | **0.003** | | | -32.5 | | | (-69.0 to 32.5) | | 0.26 | | | |  |
| Measles |  | |  |  | | |  |  | | |  | | |  | | |  | | |  | |  | | | |  |
| Male | 34 | | 132 (8.9) | 57 | | | 298 (9.0) | 166 | | | (-17 to 637) | | | 0.092 | | | 205 | | | (-26 to 831) | | 0.11 | | | |  |
| Female | 24 | | 163 (9.3) | 57 | | | 227 (9.3) | 64 | | | (-84 to 486) | | | 0.54 | | | 94 | | | (-79 to 590) | | 0.41 | | | |  |
| MV | 33 | | 145 (9.4) | 49 | | | 274 (9.4) | 128 | | | (-48 to 627) | | | 0.23 | | | 143 | | | (-46 to 649) | | 0.20 | | | |  |
| MV+DTP | 25 | | 144 (8.6) | 65 | | | 242 (9.0) | 98 | | | (-53 to 500) | | | 0.30 | | | 165 | | | (-57 to 805) | | 0.22 | | | |  |

**Supplementary Table 2 Effect of HCMV infection on cytokine levels in unstimulated whole blood**

Whole blood was incubated for 18 hours with no antigen stimulus added as a measure of background cytokine production.

^1^Estimated using general linear modelling. ^2^Covariates selected for adjustment by forward stepwise regression as described in methods. N = number of infants analysed, Geo mean = geometric mean, GSD = geometric standard deviation, Δ = mean difference, 95% CI = 95% confidence interval.

|  | CMV- | | | CMV+ | | | Comparison^1^ (unadjusted) | | | Comparison^1^ (adjusted) ^2^ | | |
| --- | --- | --- | --- | --- | --- | --- | --- | --- | --- | --- | --- | --- |
|  | N | Geo Mean (GSD) | | N | Geo Mean (GSD) | | Δ | 95%CI | P-value | Δ | 95%CI | P-value |
| IL-4 | 48 | | 3.56 (4.37) | 98 | | 4.57 (5.44) | 1.01 | (-1.11 to 4.97) | 0.43 | -0.98 | (-2.69 to 1.66) | 0.41 |
| IL-1β | 48 | | 52 (12.9) | 98 | | 103 (26.5) | 52 | (-18 to 268) | 0.23 | -39 | (-85 to 40) | 0.27 |
| IL-10 | 48 | | 74 (9) | 98 | | 171 (15) | 98 | (-7 to 368) | 0.081 | 45 | (-35 to 170) | 0.32 |
| IL-12(p70) | 48 | | 49.2 (6.6) | 98 | | 44.9 (5.3) | -4.3 | (-27.7 to 44.4) | 0.81 | -2.4 | (-17.6 to 21.9) | 0.81 |
| Eotaxin | 48 | | 201 (5.3) | 98 | | 273 (4.7) | 72 | (-59 to 325) | 0.36 | 3 | (-106 to 179) | 0.97 |
| GMCSF | 48 | | 0.85 (8.7) | 98 | | 3.00 (18.1) | 2.15 | (0.28 to 7.09) | **0.011** | 1.87 | (-0.12 to 6.44) | 0.075 |
| IFN-γ | 48 | | 580 (7.7) | 98 | | 904 (7.4) | 324 | (-182 to 1471) | 0.29 | -17 | (-423 to 717) | 0.95 |
| PDGFBB | 48 | | 5752 (3.3) | 98 | | 6975 (3.1) | 1223 | (-1389 to 5399) | 0.42 | 743 | (-1154 to 3372) | 0.49 |
| TNF | 48 | | 38 (16) | 98 | | 115 (25) | 77 | (-2 to 333) | 0.064 | 38 | (-23 to 145) | 0.27 |
| VEGF | 48 | | 302 (7.5) | 98 | | 231 (9.5) | -71 | (-203 to 235) | 0.53 | -67 | (-178 to 122) | 0.41 |
| TNF/IL-10 | 48 | | 0.53 (4.47) | 98 | | 0.67 (5.13) | 0.15 | (-0.17 to 0.73) | 0.44 | 0.11 | (-0.20 to 0.70) | 0.56 |
| IFN-γ/IL-10 | 48 | | 8.08 (8.37) | 98 | | 5.25 (9.82) | -2.82 | (-5.90 to 4.58) | 0.34 | -0.29 | (-3.03 to 5.95) | 0.89 |
| IFN-γ/IL-4 | 48 | | 164 (4.6) | 98 | | 197 (5.5) | 33 | (-60 to 209) | 0.58 | 26 | (-71 to 219) | 0.68 |

**Supplementary Table 3 Effect of HCMV infection on cytokine levels in unstimulated whole blood**

Whole blood was incubated for 18 hours with the measles peptide pool for measles vaccinated infants (MV and MV+DTP groups) or tetanus toxoid for DTP vaccinated infants (DTP or MV+DTP groups). Table shows only those cytokine / stimulus combinations which showed significant differences between HCMV+ and HCMV- infants. ^1^Estimated using general linear modelling. ^2^Covariates selected for adjustment by forward stepwise regression as described in methods. N = number of infants analysed, Geo mean = geometric mean, GSD = geometric standard deviation, Δ = mean difference, 95% CI = 95% confidence interval.

|  | **CMV-** | | | **CMV+** | | | | **Comparison^1^ (unadjusted)** | | | | | | | | | **Comparison^1^ (adjusted) ^2^** | | | | | | | | |  |
| --- | --- | --- | --- | --- | --- | --- | --- | --- | --- | --- | --- | --- | --- | --- | --- | --- | --- | --- | --- | --- | --- | --- | --- | --- | --- | --- |
|  | | **N** | **Geo Mean (GSD)** | | **N** | | **Geo Mean (GSD)** | | **Δ** | | | **95%CI** | | | **P-value** | | | **Δ** | | | **95%CI** | | | **P-value** | | |
| Measles Pool Stimulated Eotaxin | | | |  | |  | |  | |  | | |  | | |  | | |  | | | |  | |  |  |
| MV | 17 | | 112 (7.2) | 34 | | | 311 (6.0) | 198 | | | (27 to 583) | | | 0.013 | | | 106 | | | (-5 to 320) | | 0.067 | | | |  |
| MV+DTP | 16 | | 299 (4.2) | 38 | | | 211 (4.2) | -88 | | | (-228 to 327) | | | 0.53 | | | -172 | | | (-247 to -40) | | **0.017** | | | |  |
| Measles Pool Stimulated VEGF | | | |  | | |  |  | | |  | | |  | | |  | | |  | |  | | | |  |
| MV | 17 | | 342 (6.7) | 34 | | | 192 (8.6) | -150 | | | (-267 to 150) | | | 0.23 | | | -117 | | | (-198 to 32) | | 0.10 | | | |  |
| MV+DTP | 16 | | 407 (3.4) | 38 | | | 256 (11.1) | -151 | | | (-324 to 387) | | | 0.42 | | | -330 | | | (-440 to -89) | | **0.016** | | | |  |
| Tetanus Toxoid Stimulated IL-1β | | | |  | | |  |  | | |  | | |  | | |  | | |  | |  | | | |  |
| DTP | 12 | | 543 (17) | 30 | | | 808 (29) | 265 | | | (-427 to 5096) | | | 0.69 | | | 116 | | | (-100 to 656) | | 0.40 | | | |  |
| MV+DTP | 16 | | 942 (27) | 38 | | | 653 (19) | -289 | | | (-832 to 2953) | | | 0.69 | | | -321 | | | (-416 to -63) | | **0.03** | | | |  |
| Tetanus Toxoid Stimulated Eotaxin | | | |  | | |  |  | | |  | | |  | | |  | | |  | |  | | | |  |
| DTP | 12 | | 77 (6.7) | 30 | | | 109 (3.7) | 32 | | | (-41 to 262) | | | 0.54 | | | 28 | | | (-38 to 198) | | 0.54 | | | |  |
| MV+DTP | 16 | | 102 (4.6) | 38 | | | 324 (5.2) | 223 | | | (34 to 675) | | | **0.009** | | | 86 | | | (4 to 234) | | **0.035** | | | |  |
| Tetanus Toxoid Stimulated TNF | | | |  | | |  |  | | |  | | |  | | |  | | |  | |  | | | |  |
| DTP | 12 | | 409 (12) | 30 | | | 474 (20) | 64 | | | (-324 to 2219) | | | 0.87 | | | 21 | | | (-130 to 410) | | 0.85 | | | |  |
| MV+DTP | 16 | | 316 (12) | 38 | | | 1148 (22) | 832 | | | (-58 to 4789) | | | 0.090 | | | 231 | | | (46 to 614) | | **0.005** | | | |  |
